# Supplementary material for: Estimating the Spatial Distribution and Future Conservation Requirements of the Spotted Seal in the North Pacific
Source: Animals (Basel). 2023 Oct 19;13(20):3260. doi: 10.3390/ani13203260 (PMC10603672; doi:10.3390/ani13203260)
Supplement: Supplementary file 1 [file animals-13-03260-s001.zip › animals-2624908-SI.pdf]

Supplementary material

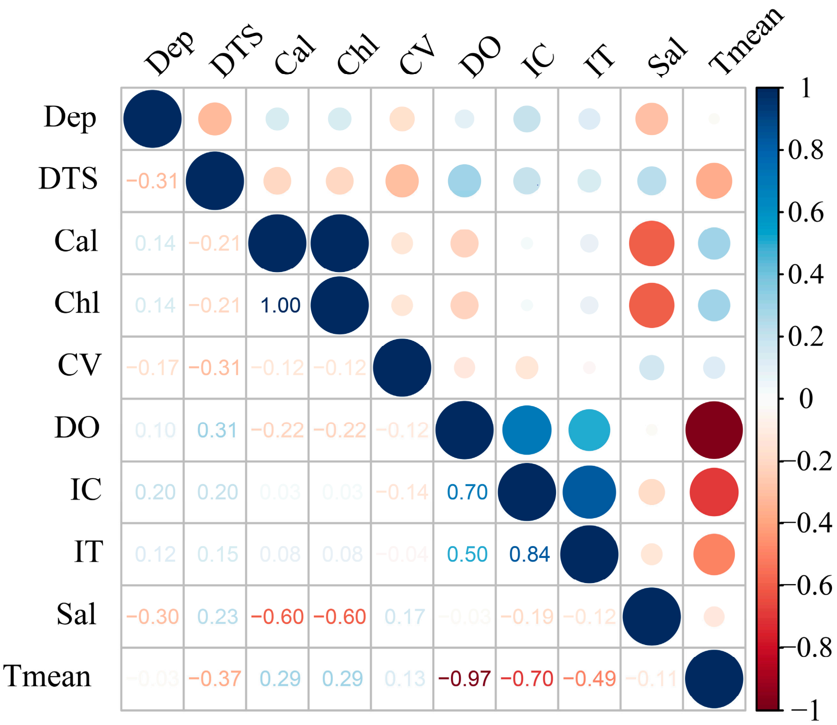

Supplementary Figure S1 Results of Pearson correlation analysis for 10 environmental variables.

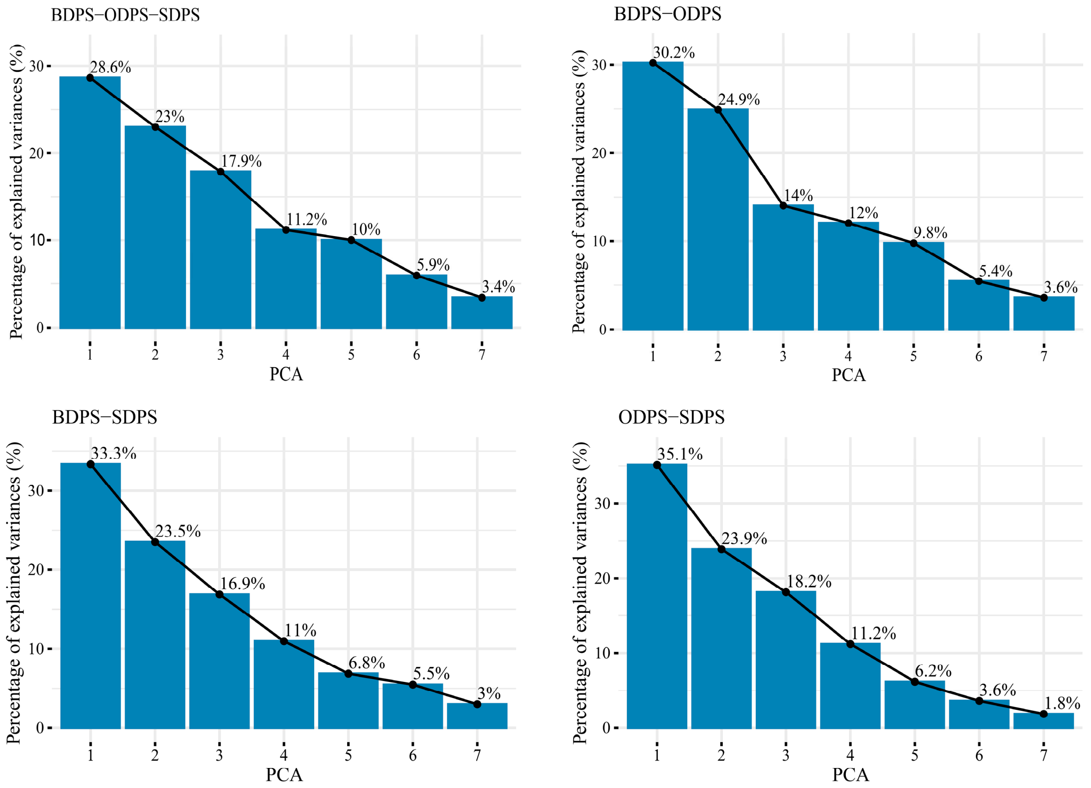

Supplementary Figure S2 The results of principal component analyses based on all the environmental variables involved in the model using the R package FactoMineR. The number in the figure indicates the percentage of explained variance for each principal component.

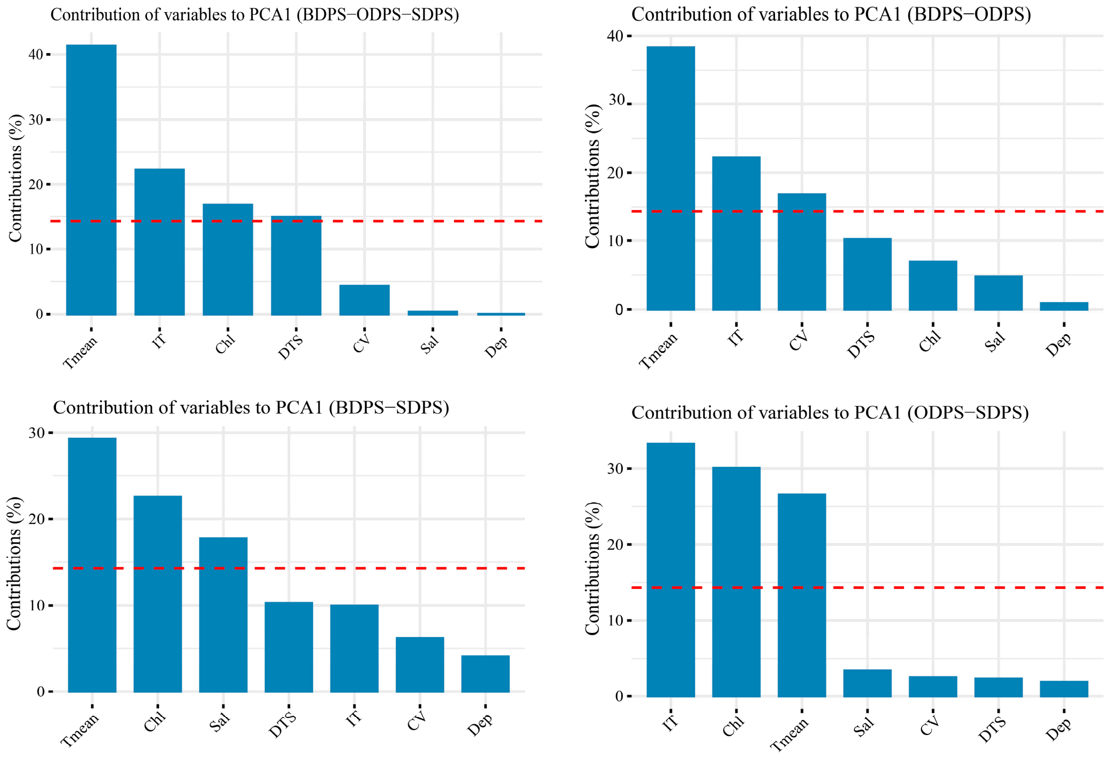

Supplementary Figure S3 The degree of contribution of environmental variables to the principal component which has the greatest influence on ecological niche differentiation between populations. Dep, water depth; DTS, distance to shore; Tmean, water temperature; Sal, salinity; IT, ice thickness; CV, currents velocity.

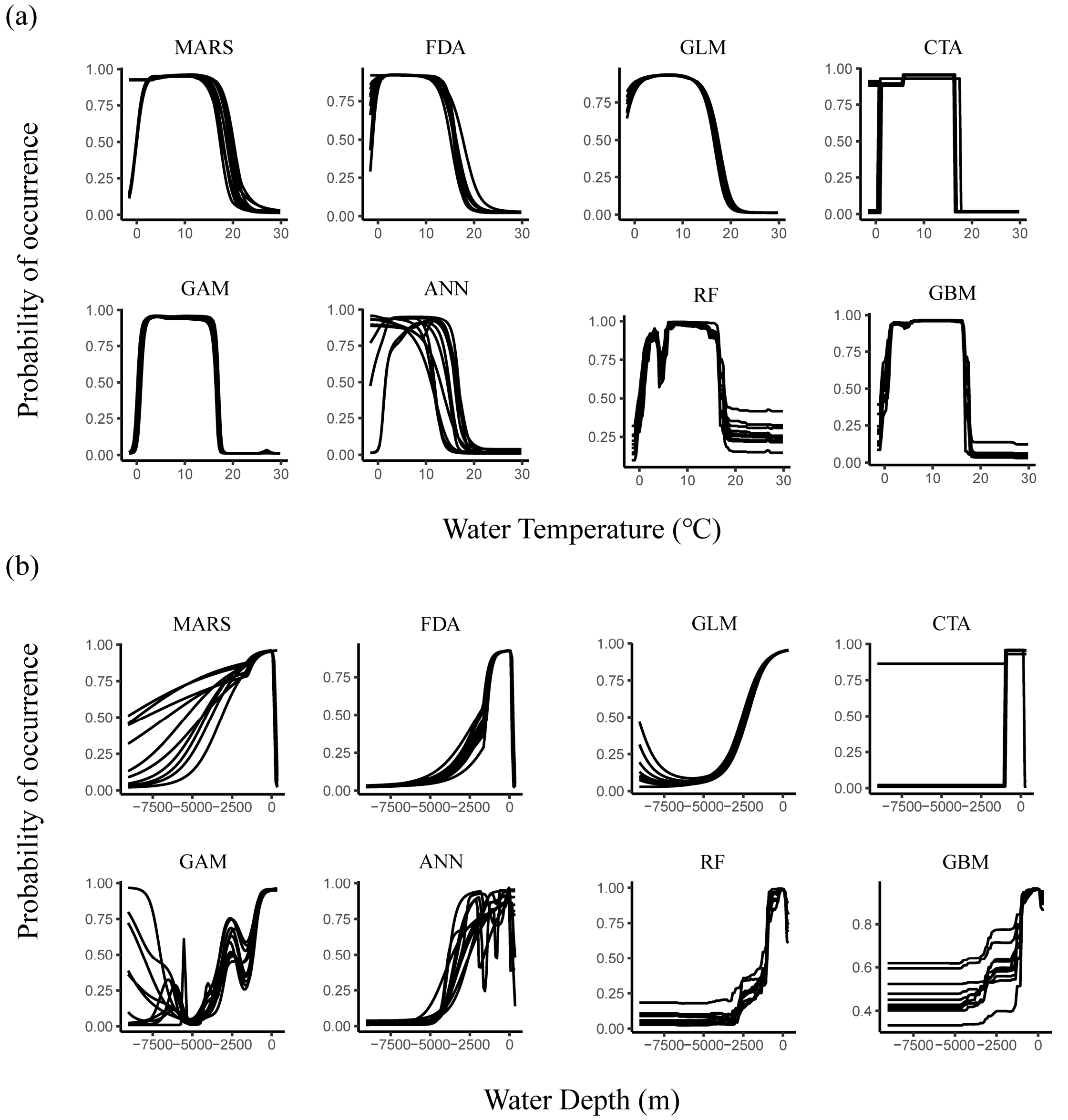

Supplementary Figure S4 The response curves of spotted seals probability of occurrence against water temperature (a) and water depth (b) based on the species-level model.

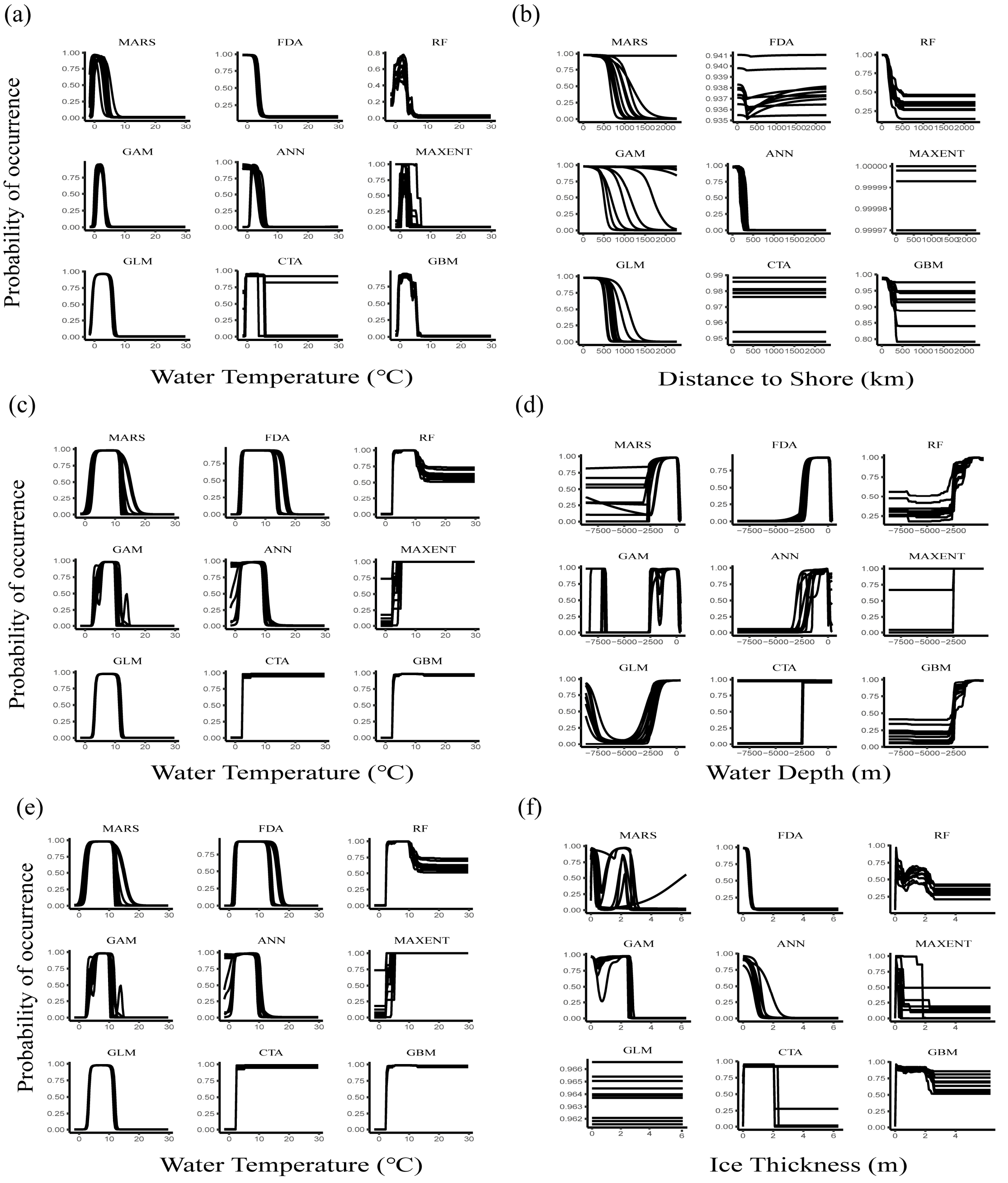

Supplementary Figure S5 The response curves of population probability of occurrence against the two most important driving factors for the Bering population (a, b), the Okhotsk population (c, d), and the Southern population (e, f) based on the population-level model.

Supplement Table S1 Reference list of occurrence data for spotted seal (*Phoca largha*) in this research.

Numbers	References	First author	Record time	Number of records
1	Migrations of young spotted seals (Phoca largha) from Peter the Great Bay, Sea of Japan/East Sea, and the pattern of their use of seasonal habitats	Alexey M. Trukhin	2017.5-2018.3	27234
2	The First Data on the Use of the Pacific Ocean Area by the Okhotsk Population of the Spotted Seal Phoca largha	M. A. Solovyeva	2017.7-2018.1	73
3	Changes in the Breeding Range of Spotted Seals, Phoca largha (Pallas, 1811) (Carnivora, Pinnipedia), in the Western Sea of Japan:Causes and Effects	Alexey M. Trukhin	2002-2017	52
4	Movements of Spotted Seals (Phoca largha) in the Sea of Okhotsk According to Satellite Tagging Data	M. A. Solov'eva	2011.10-2013.9	272
5	Release studies on spotted seals (Phoca largha) using satellite telemetry tracking technique	HAN Jiabo	2010-2011	646
6	Distribution and abundance of spotted seals Phoca largha and ribbon seals Phoca fasciata in the southern Sea of Okhotsk	Ayako W. Mizuno	2000.3-2000.4	57
7	Habitat use and habitat selection by spotted seals (Phoca largha) in the Bering Sea	L.F. Lowry	1991.11-1994.6	120
8	Movements and behavior of satellite-tagged spotted seals (Phoca largha) in the Bering and Chukchi Seas	Lloyd F. Lowry	1991.8-1993.11	132
